# Impact of the 237th Residue on the Folding of Human Carbonic Anhydrase II

**DOI:** 10.3390/ijms12052797

**Published:** 2011-04-28

**Authors:** Ming-Jie Wu, Yan Jiang, Yong-Bin Yan

**Affiliations:** 1 Key Laboratory of Bio-Resources and Eco-Environment of MOE, College of Life Science, Sichuan University, Chengdu 610064, China; E-Mail: wmj213@gmail.com; 2 State Key Laboratory of Biomembrane and Membrane Biotechnology, Department of Biological Sciences and Biotechnology, Tsinghua University, Beijing 100084, China

**Keywords:** human carbonic anhydrase II, carbonic anhydrase II deficiency syndrome (CADS), guanidine hydrochloride-induced unfolding, FoldX

## Abstract

The deficiency of human carbonic anhydrase II (HCAII) has been recognized to be associated with a disease called CAII deficiency syndrome (CADS). Among the many mutations, the P237H mutation has been characterized to lead to a significant decrease in the activity of the enzyme and in the Gibbs free energy of folding. However, sequence alignment indicated that the 237th residue of CAII is not fully conserved across all species. The FoldX theoretical calculations suggested that this residue did not significantly contribute to the overall folding of HCAII, since all mutants had small ΔΔ*G* values (around 1 kcal/mol). The experimental determination indicated that at least three mutations affect HCAII folding significantly and the P237H mutation was the most deleterious one, suggesting that Pro237 was important to HCAII folding. The discrepancy between theoretical and experimental results suggested that caution should be taken when using the prediction methods to evaluate the details of disease-related mutations.

## 1. Introduction

The successful folding to its native structure ensures that the protein functions correctly, while the appropriate stability determines the life cycle of the protein in the cell. When the protein is incorrectly folded or prone to be misfolded, the aberrant structure may lead to loss-of-function or gain-of-function of proteins, which has been associated to many serious diseases [1]. Particularly, many familial conformational diseases are caused by single point mutations [2]. Thus the elucidation of how these disease-related mutations affect the structure and stability of proteins not only helps us to discover the molecular mechanism of the corresponding disease, but also facilitates our understanding of the structure-function relationship of these proteins. To evaluate the mutations on protein stability quantitatively, two possible ways are available: one is by experimental work (for example, [3–8]) and the other is by prediction (for example, [9–14]).

**Figure 1.**
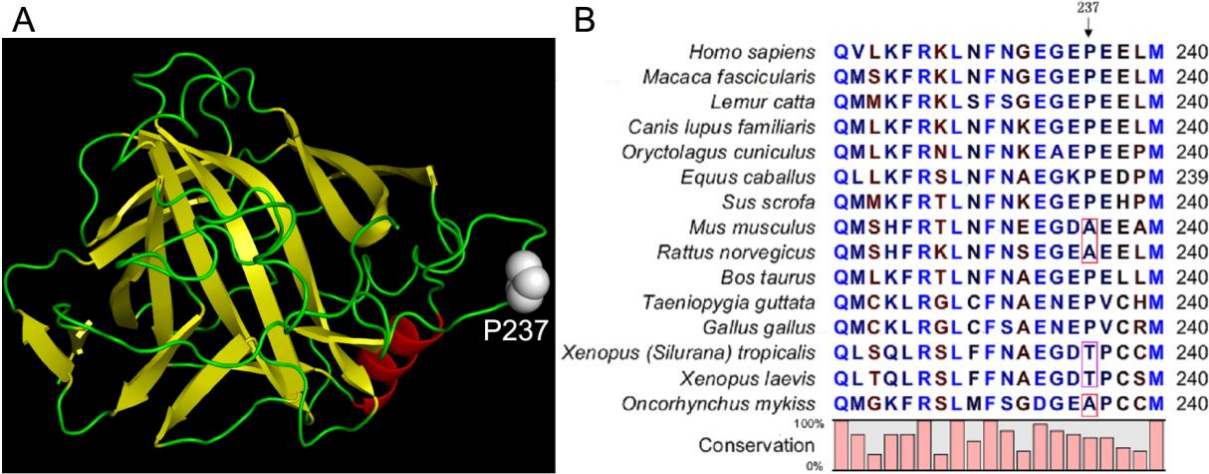
(**A**) Crystal structure of HCAII (PDB ID 2CBA). Pro237 is highlighted by space-filling model; (**B**) Sequence alignment of CAII.

Carbonic anhydrase (CA), a member of a large zinc metalloenzyme family, catalyzes the reversible reaction in which carbondioxide is hydrated into bicarbonate [15]. In higher vertebrates, there are several CA isoenzymes with dissimilar cellular and tissue distributions [15,16]. The deficiency of human CA II (HCAII) has been recognized to be associated with a disease called CAII deficiency syndrome (CADS) [17]. Particularly, the deficiency of HCAII can be caused by single-point mutations [16,18], which may severely affect HCAII catalytic properties and stability [4,5,19]. Among these mutations, the P237H mutation was found to significantly decrease the catalytic efficiency and stability of HCAII, suggesting that this mutation may lead to loss-of-function of HCAII and further result in HCAII deficiency [5]. P237 is located on the surface of the HCAII molecule (Figure 1), and is not involved in the active site or core structure of the enzyme. However, the dramatic decrease in the reduction of the Gibbs free energy of HCAII folding implied that it might be crucial to the correct packing of the adjacent secondary structures [5].

In this research, multiple sequence alignment was performed to check whether the 237th residue is highly conserved across species. Surprisingly, the sequence alignment indicated that the 237th residue is not fully conserved. It is Ala in CAs from *Mus musculus*, *Rattus norvegicus* and *Oncorhynchus mukiss*, and Thr in CAs from *Xenopus tropicalis* and *Xenopus laevis* (Figure 1). To investigate the role of the 237th residue in HCAII structure and folding, the effect of substitutions of Pro by various amino acid residues at position 237 was studied by both FoldX prediction and folding experiments. The results indicated that all mutations caused a minor decrease of the Gibbs free energy of HCAII stability when evaluated by FoldX prediction. The experimental results were consistent with the prediction for most mutations except P237H. Since the mutations mainly affect the stability of the molten globular intermediate, it might be difficult for the algorithm to predict the changes of the Gibbs free energy of non-native states of proteins.

## 2. Results and Discussion

### 2.1. Stability Changes by Mutations of the 237th Residue Predicted by FoldX

The effect of the disease-related mutation P237H on HCAII folding has been investigated previously, and it was found that this mutation led to a ∼7.3 kcal/mol decrease of HCAII stability [5]. However, sequence alignment analysis indicated that the 237th residue is not fully conserved in CAII, and Ala and Thr also appear in the CAII sequence from the other species (Figure 1). An unresolved question is whether the position 237 of CAII has any amino acid residue preference? To elucidate this problem, the mutation-induced stability changes were evaluated by the prediction of FoldX [20], a well-established method that has been successfully applied to the analyses of protein folding [14,21], protein design [22], protein-protein interactions [23,24], protein-DNA binding [25] and evolution [26] in a variety of proteins. The prediction was carried out using the standard procedures, and the “RepairPDB” command was performed before calculation to minimize the FoldX free energy for the WT structure at 25 °C.

**Figure 2.**
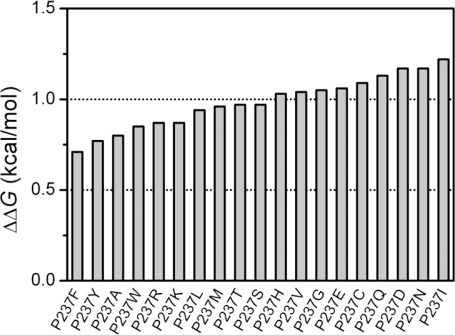
The changes in the Gibbs free energy induced by mutations predicted by FoldX.

As shown in Figure 2, all the mutations tested had small ΔΔ*G* values around 1 kcal/mol, and the largest change in stability was found to be caused by the P- > I mutation with a value of 1.22 kcal/mol. These results suggest that according to the FoldX prediction, the substitution of Pro at position 237 by any of the other residues did not significantly affect HCAII stability. In other words, Pro237 contributed little to the overall stability of the protein. The large discrepancy between the experimental data (ΔΔ*G* = 7.3 kcal/mol) and the prediction (ΔΔ*G* = 1.03 kcal/mol) of the P237H mutation suggested that the role of Pro237 in HCAII might not be well evaluated by FoldX. In this case, it is necessary to determine the changes in Gibbs free energy by experimental methods. Five typical mutations (P237A, P237T, P237N, P237I, P237F) were chosen for further analysis by folding studies. The choice of P237A and P237T was due to the appearance of these two residues in the sequence of the other species (Figure 1). The other three mutations were chosen because according to the FoldX results shown in Figure 2, P237F was the most stable one among the mutants, while P237I and P237 N were the most unstable ones among the possible 20 natural amino acids.

### 2.2. Characterization of the Mutants

**Figure 3.**
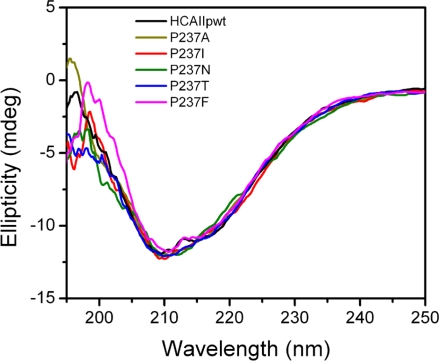
Far-UV CD spectra of HCAII_pwt_ and the mutants.

**Figure 4.**
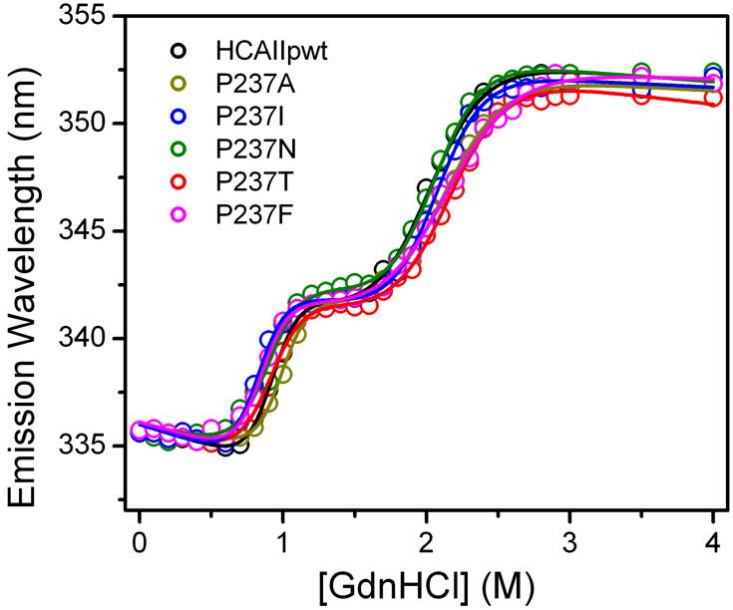
Unfolding transition curves of HCAII_pwt_ and the mutants monitored by the maximum wavelength of the intrinsic Trp fluorescence. The raw data were fitted by a three-state model and presented as solid lines.

HCAII_pwt_, which contains a C206S mutation to avoid the interference of unexpected disulfide formation, was used in this study. Previous studies have shown that HCAII_pwt_ have indistinguishable folding and functional properties from the wild type protein [27–31]. All recombinant proteins could be successfully obtained in the soluble fraction when overexpressed in *E. coli*. The activities of the mutants were similar to HCAII_pwt_, ranging from 87% to 99% of the activity of HCAII_pwt_ (Table 1). The effect of the mutations on HCAII structure was investigated by circular dichrosim (CD) (Figure 3) and intrinsic fluorescence (data not shown, see also Figure 4) experiments. The spectra of the mutants were almost superimposed with those of HCAII_pwt_, suggesting that the mutations did not affect either the secondary or the tertiary structures of HCAII_pwt_.

### 2.3. Evaluation of the Stability Changes by GdnHCl-Induced Folding Experiments

The HCAII sequence contains seven Trp residues distributed throughout the folded protein structure, thus the conformational changes of the proteins can be sensitively monitored by intrinsic Trp fluorescence. The emission maximum wavelength of the intrinsic Trp fluorescence was measured at each GdnHCl concentration tested, and the results are presented in Figure 4. Consistent with previous observations [5,27,28], the transition curves of all proteins were a three-state process with a molten globular intermediate state (I) appearing between the native (N) and the unfolded (U) state. By fitting the transition curves into the three-state model N↔I↔U, the Gibbs free energy of the two transitions was obtained, and the changes in stability (ΔΔ*G*) were calculated accordingly for each mutant (Table 1). The Δ*G* values of HCAII_pwt_ were similar to those in literature [4,5], and the minor deviations might be caused by different experimental procedures.

**Figure 5.**
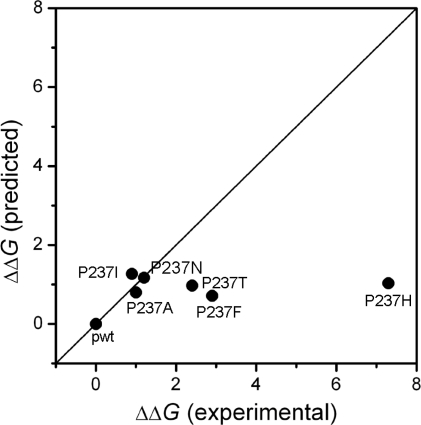
Correlation between the FoldX prediction and the experimental measurements.

Most mutations slightly destabilize both the N↔I and I↔U transitions except P- > A mutation seems to stabilize the N↔I transition. The ΔΔ*G* values of folding were between 0.9 and 2.9 kcal/mol. The most destabilized mutation was P- > F, while the least was P- > I. This observation was quite different from the FoldX prediction, which indicated that HCAII_P237I_ was the most unstable and HCAII_P237F_ was the most stable mutant. The large discrepancy between the theoretical and experimental ΔΔ*G* values for the three mutants HCAII_P237T_, HCAII_P237F_ and HCAII_P237H_ (Figure 5) suggested that the effects of these mutations could not be predicted correctly. One possible reason is that the FoldX has a correlation coefficient of 0.81 and a standard deviation of 0.46 kcal/mol [20], and another may be that the prediction can give reasonable data of a large data set but not for the details of a small set, as indicated by other authors [32]. Nonetheless, the large experimental ΔΔ*G* values (>2 kcal/mol) caused by the P237T, P237F and P237H mutations implied that the position 237 of CAII should play a role in CAII stability, and the disease-related mutation P237H was the most deleterious.

## 3. Experimental Section

### 3.1. Materials

Tris, sulfuric acid, ultra pure guanidine hydrochloride (GdnHCL), p-nitrophenol acetate(p-NPA) and isopropyl-β-d-thiogalactopyranoside (IPTG) were purchased from Sigma. All the other chemicals were local products of analytical grade.

### 3.2. Site-Directed Mutagenesis

According to the previous report, a pseudo wild type HCAII (HCAII_pwt_) was used in this research. This pseudo wild type protein was constructed with the mutation C206S to avoid possible interference from the folding of HCAII by incorrect disulfide formation since Cys206 is the only Cys in HCAII. Previous study has shown that HCAII_pwt_ has the same catalytic properties and folding as the wild type HCAII [27]. The mutated proteins were obtained by site directed mutagenesis using the following primers:

HCAII_P237I_-For, 5′-CAATGGGGAGGGTGAA**ATC**GAAGAACTGATG-3′;
HCAII_P237I_-Rev, 5′-CATCAGTTCTTC**GAT**TTCACCCTCCCCATTG-3′;
HCAII_P237N_-For, 5′-CAATGGGGAGGGTGAA**AAC**GAAGAACTGATG-3′;
HCAII_P237I_-Rev, 5′-CATCAGTTCTTC**GTT**TTCACCCTCCCCATTG-3′;
HCAII_P237F_-For, 5′-CAATGGGGAGGGTGAA**TTC**GAAGAACTGATG-3′;
HCAII_P237F_-Rev, 5′-CATCAGTTCTTC**GAA**TTCACCCTCCCCATTG-3′;
HCAII_P237A_-For, 5′-CAATGGGGAGGGTGAA**GCC**GAAGAACTGATG-3′;
HCAII_P237A_-Rev, 5′-CATCAGTTCTTC**GGC**TTCACCCTCCCCATTG-3′;
HCAII_P237T_-For, 5′-CAATGGGGAGGGTGAA**ACC**GAAGAACTGATG-3′;
HCAII_P237T_-Rev, 5′-CATCAGTTCTTC**GGT**TTCACCCTCCCCATTG-3′.

Site-directed mutations were carried out using standard procedures. The genes were cloned into PET28b vector (Novagen) and a 6-His tag were added at the C-terminus of the protein to facilitate protein purification.

### 3.3. Protein Expression and Purification

HCAII_pwt_ and the mutated proteins were overexpressed in *E. coli* Rosseta(DE3) in LB_kan_ at 37 °C, and the induction of overexpression was achieved by the addition of 0.5 mM IPTG and 0.5 mM ZnSO_4_. The bacterial cells were harvested by centrifugation, sonicated and the target proteins were purified by Ni-NTA affinity chromatography (QIAGEN) as described previously [5]. The final products were collected on a Superdex 75 HR 10/30 (GE Healthcare Life Sciences), and only the peak containing the monomeric form were collected. The protein concentration were determined by measuring the absorbance at A_280_ using *ɛ*_280 nm_ = 53,800 M^−1^ cm^−1^.

### 3.4. Activity Assay

The enzymatic activity of HCAII_pwt_ and the mutants were determined by the esterase activity assay, which monitors the appearance of *p*-nitrophenolate anion spectrophototometrically during the hydrolysis of p-NPA [33]. The 1-mL assay mixtures contained 1 mM pNPA, 3% acetone and 10 mM Tris-H_2_SO_4_, pH 7.5. The reaction was started by the addition of 1.5 μM enzyme, and the hydrolysis of pNPA to pNP was monitored by following the increase in absorbance at 348 nm at 25 °C. The final value was obtained by subtracting the background values for the non-catalyzed ester hydrolysis.

### 3.5. Protein Folding Experiments

The unfolding of HCAII_pwt_ and the mutated proteins were performed by incubating the proteins in 10 mM Tris-H_2_SO_4_ buffer, pH 7.5, in the presence of various concentrations of GdnHCl for 16 h at 25 °C. Then spectroscopic experiments were performed to monitor the structural changes of the samples. The final protein concentration was 0.8 μM. The unfolding data were fitted to a three-state folding model as described previously [5].

### 3.6. Spectroscopic Measurements

The intrinsic Trp fluorescence was measured using a 1 cm path-length quartz cuvette on a Hitachi F-4500 spectrophotometer at 25 °C. The excitation wavelength was 295 nm with both the entrance and exit slits of 5 nm, and the emission spectra were collected between 300 nm and 450 nm. The Far-UV circular dichroism (CD) spectra were recorded on a Jasco-715 spectrophotometer (Tokyo, Japan) over a wavelength range of 190–250 nm using the 0.1 cm path-length cells. The protein concentration for CD experiments was 1.5 μM. The presented spectra were the average of three repetitions.

### 3.7. Changes in Gibbs Free Energy Calculated by FoldX

The changes in the Gibbs free energy (ΔΔ*G*) induced by mutations at position 237 were calculated by FoldX (version 3.0 beta3) [20]. The structure of the wild type protein (PDB ID: 2CBA) was minimized using the “RepairPDB” command to identify the residues that had bad torsion angles, van der Waal’s clashes or total energies belonging to the complex interface. Then individual mutations were built using “BuildModel” command and the ΔΔ*G* values were extracted from the FoldX output files.

## 4. Conclusions

Sequence alignment indicated that the 237th residue of CAII is not fully conserved across all species. However, the P237H mutation of HCAII has been characterized to be a disease-related mutation that significantly destabilizes the protein. The FoldX theoretical calculations suggested that this residue did not significantly contribute to the overall folding of HCAII since a small ΔΔ*G* value (around 1 kcal/mol) was obtained when substituting Pro237 by any other naturally occurring amino acid. The experimental studies indicated that at least three mutations significantly affected the GdnHCl-induced unfolding of HCAII, suggesting that Pro237 is important to HCAII folding. The results also showed that the P237H mutation was the most deleterious among the 19 mutations. The discrepancy between theoretical and experimental results suggested that caution should be taken when using the prediction methods to evaluate the details of disease-related mutations.

## Figures and Tables

**Table 1.** Relative activity and the thermodynamic parameters of HCAII_pwt_ and the mutants. Δ*G* and ΔΔ*G* are presented in kcal/mol, and m is in kcal mol^−1^ M^−1^ (GdnHCl). The ΔΔ*G* values were obtained by subtracting the Δ*G* values of HCAII_pwt_ from that of the mutants. The activity of the enzymes was normalized by taking HCAII_pwt_ as 100%.

**Enzyme**	**Δ*G*_NI_**	**Δ*G*_IU_**	**Δ*G*_NU_**	**ΔΔ*G*_NU_**	***m*_NI_**	***m*_IU_**	**Activity(%)**
HCAII_pwt_	5.7 ± 0.6	7.8 ± 0.6	13.5	0	6.3 ± 0.6	3.9 ± 0.2	100
HCAII_P237A_	6.5 ± 0.7	6.0 ± 0.4	12.5	1.0	6.5 ± 0.7	2.8 ± 0.2	87
HCAII_P237T_	4.7 ± 0.5	6.4 ± 0.4	11.1	2.4	5.2 ± 0.6	3.0 ± 0.2	99
HCAII_P237N_	5.0 ± 0.7	7.3 ± 0.5	12.3	1.2	5.5 ± 0.8	3.6 ± 0.3	93
HCAII_P237I_	5.1 ± 0.5	7.5 ± 0.4	12.6	0.9	6.2 ± 0.6	3.7 ± 0.2	91
HCAII_P237F_	5.3 ± 0.6	5.3 ± 0.3	10.6	2.9	6.3 ± 0.7	2.5 ± 0.1	91
HCAII_P237H_	−	−	−	7.3	−	−	−
